# Age-friendly city: future perspectives for the Brazilian cities

**DOI:** 10.1590/1980-57642021dn15-030001

**Published:** 2021

**Authors:** Patricia de Oliveira Lopes, Simone Rezende da Silva, Tathianni Cristini da Silva, Yara Dadalti Fragoso, Angelina Zanesco

**Affiliations:** 1 Postgraduate Program in Environmental Health, Universidade Metropolitana de Santos – Santos, SP, Brazil.

**Keywords:** elderly, aging, World Health Organization, United Nations, idosos, envelhecimento, Organização Mundial da Saúde, Nações Unidas

## Abstract

The world population is aging fast and not all cities are prepared to cope with the needs of the elderly people. Cities need to develop strategies for senior citizens including the aspects of health, nutrition, consumer protection, housing, transportation, environment, social welfare, income, employment, safety, and education. The World Health Organization (WHO) created a program dedicated to older adults called the *age-friendly city*. This program is about creating the environment and opportunities that enable older people to be and do what they value throughout their lives. Most of the elderly population lives in urban spaces, and aging represents a challenge as well as opportunities to the cities all over the world. Recently, only 16 Brazilian cities have received the seal of international certification by meeting the requirements stipulated by the WHO. In the State of Sao Paulo, only two cities have been qualified for this seal. Therefore, the aims of this article are (a) to provide a brief history of this important initiative taken by the WHO and (b) to urge the decision-makers of Brazilian municipalities to develop effective initiatives for their cities to be prepared for this demographic modification.

## RESUMO

A população mundial está envelhecendo rapidamente, e nem todas as cidades estão preparadas para lidar com as necessidades dessa população. As cidades devem desenvolver estratégias para os idosos, incluindo aspectos de saúde, nutrição, defesa do consumidor, habitação, transporte, meio ambiente, assistência social, renda, emprego, segurança e educação. A Organização Mundial da Saúde (OMS) possui um programa denominado “cidade amiga dos idosos”, cujos objetivos são preparar as cidades para o envelhecimento da população e minimizar os custos desse processo proporcionando a inserção ativa dos idosos na vida socioeconômica das cidades. A certificação (selo) de cidade amiga dos idosos pela OMS. A maioria da população idosa vive nos espaços urbanos, o que isso representa um desafio e um ambiente de oportunidades para as cidades. Um desafio, pois ao envelhecer ocorre uma maior demanda pelos cuidados em saúde e instituições de longa permanência. Por outro lado, a população idosa apresenta um potencial de ambiente de negócios específicos para essa faixa etária, como os setores de alimentação, educação, e setores de entretenimento. No Brasil, a população idosa responde por cerca de 20% do consumo nacional. Apenas 16 cidades brasileiras receberam esse selo de certificação internacional para atender aos requisitos estipulados pela OMS. No estado de São Paulo, apenas duas cidades foram qualificadas para a obtenção do selo. Portanto, os objetivos do presente artigo são a) fornecer um breve histórico desse importante programa da OMS; b) despertar nos gestores das cidades a vontade de desenvolver iniciativas efetivas para que as cidades estejam preparadas para um rápido envelhecimento populacional, que tenha potencial para contribuir com a sociedade em suas diferentes capacidades.

## A BRIEF HISTORY OF AN AGE-FRIENDLY CITY PROGRAM

The aging of the world population is a factor for social transformation in this century. According to the United Nations (UN), about 71 million elderly people existed in the world in 2000, and this number had increased to 600 million by 2017. The elderly population in the world is expected to reach 2 billion in 2050.^1^ The UN has implemented debates on protection and action programs aiming the elderly population *via* the World Health Organization (WHO). The evolution of these programs is described in detail below.

In 1948, the rights of the elderly people were first discussed at the UN. Almost 30 years later, in the 1970s, this issue was again raised, highlighting the need for urgent measures since life expectancy was increasing.^2^ However, it was not until 1982 that the UN convened its members to the first UN General Assembly at which aging was discussed. On that occasion, the Vienna International Plan of Action on Aging was outlined (Resolution 37/5).^3^ In this plan, 62 recommendations were established for senior citizens, involving health, nutrition, protection of consumers, housing and transportation, environment, family issues, social welfare, income, and employment security and education. Research data involving different areas of knowledge had been collected and analyzed to enable a better understanding of the aging process. In that document, the UN emphasized that longevity was an achievement of the biological process and the improvement of populations’ health, considering “elderly” to be a person aged 60 years or above.^3^

The establishment of a “seal” for age-friendly cities around the world came as an advance through the challenges and achievements of Resolution 37/5. Cities, certified as age-friendly, need to have demonstrated their provision for social participation and inclusion, employment, community support, healthcare, housing, transportation, open spaces, communication, and information for their elderly citizens.^4^

**Figure 1.**
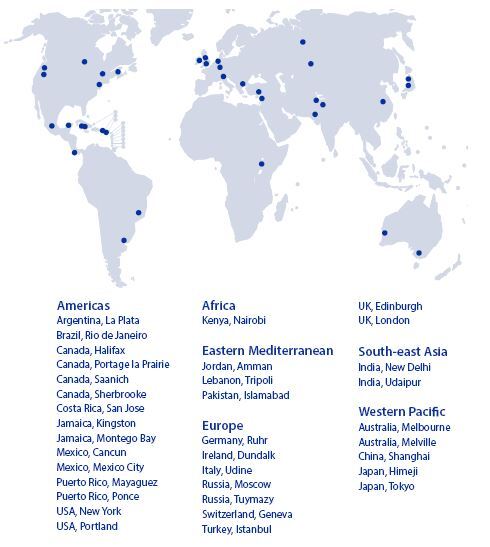
World map of the age-friendly cities leading project.

Source: World Health Organization.6

During September 2006 to April 2007, 35 cities from developed and developing countries were enrolled in the WHO project leading, including six mega-cities (i.e., Mexico City, Moscow, New Delhi, Rio de Janeiro, Shanghai, and Tokyo) with over 10 million inhabitants, “almost mega-cities” such as Istanbul, London, and New York, as well as national capitals, regional centers, and small cities. Figure 1 shows all the cities that participated at the beginning of the age-friendly city program.^5,6^

**Figure 2.**
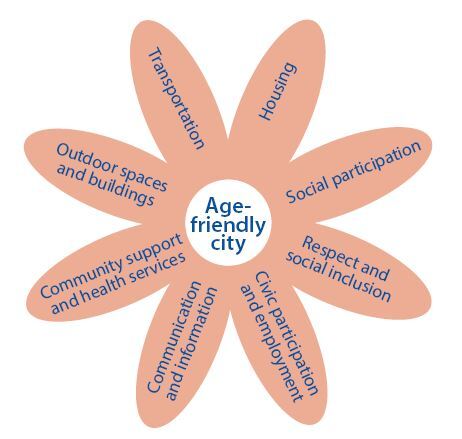
Age-friendly city domains for rating the cities all over the world.

A total of eight domains were established as a guide where the cities and communities can address to better adapt their structures and services to the needs of elderly people. Figure 2 illustrates all the domains that should be covered by the cities to get the seal by the WHO.^4,6^

**Figure 3.**
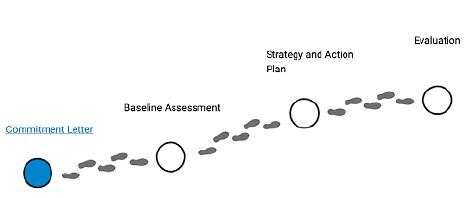
Age-friendly journey.

The process begins with a commitment letter issued by the city applying for the seal. The proposed plan of the city for meeting the conditions for receiving the seal is followed up by the WHO or its representative in the region where the city is located. After a baseline assessment, strategies are drawn up and developed. An evaluation of the city is then carried out and the seal of an age-friendly city will be given, depending on the achievements and fulfillment of requirements for such certification. Figure 3 illustrates the step of the process to apply for the seal and to continue as a member of the age-friendly city program conducted by the WHO.

At present, approximately 1,215 cities and communities have received the seal as an age-friendly city from 44 countries. In the Latin America, only nine countries received the seal from the WHO from a total of 20 countries that belong to this geographical area, about 20% of the cities that are in the program worldwide. Table 1 summarizes the countries from Latin America, the cities/communities that have the seal, and the total population of each country.^6^

**Table 1.** Age-friendly cities from Latin-American countries.

Country	Number of age-friendly cities	Total population (millions)
Argentina	15	45
Brazil	18	212
Chile	161	19.5
Colombia	01	51
Costa Rica	13	4.5
Cuba	02	11
Mexico	23	127
Peru	01	33
Uruguay	01	3.5

The progress and evaluation reports of each city are on the website of the WHO (https:/extranet.who.int/agefriendlyworld/search-assessments/?sft_countries), and it is based on the resident feedback and annual reporting to the council. According to the elderly population, the cities did not reach the goal as an age-friendly city, approximately 55% of the complaints are related to the health services, outdoor spaces (i.e., maintenance and condition of sidewalks, preservation of parks, and safety for walking in public spaces), and lack of intergenerational interaction. However, the beneficial effects of this program are certainly expected but it takes time. In fact, a recent study showed a significant slowdown in medical spending growth among the elderly people mainly for cardiovascular diseases, dementia, and aftercare for people with acute illnesses.^7^

## AGE-FRIENDLY CITIES IN BRAZIL

Taking into consideration the territorial size of Brazil and its number of inhabitants (Table 1), this country has a small number of age-friendly cities. Only 18 cities have received the seal of international certification by meeting the requirements stipulated by the WHO. Among the States of the Federation, there are age-friendly cities in the Rio Grande do Sul (3), Paraná (13), Santa Catarina (1), and São Paulo (2). On the other hand, Chile has 161 cities, Argentina has 15 cities, and Mexico has 23 cities, accredited by the WHO (2021).^5^ Particularly in Sao Paulo State, only two age-friendly cities, namely, Jaguariuna and Sao Jose do Rio Preto, have received the seal from the WHO. Since 2012, the government of the State of São Paulo, through its Secretariat of Social Development, has been working to increase the number of cities that qualify for the seal from the WHO. In 2019, about 141 centers focusing on care for the elderly people were inaugurated, with 63 day-care centers and 78 units of sheltered housing for elderly people. Considering that this state has 645 municipalities, their mayors must develop more effective initiatives, so that their cities are prepared for a growing population of elderly people. These age citizens have the potential to contribute to society in many different capacities.

The Brazilian Federal Government, through the Ministry of Health and the National Health System, has improved the collection of data from the elderly population, using the health booklet for the elderly people. The creation of this booklet had the strategic objective of assisting in health management for this age group. The material enables the recording and monitoring of information (for 5 years), such as personal, social, and family data, in addition to the health conditions of the elderly individuals and their life habits.^8^

Aging is not the same for everyone. There are determinants of individual characteristics (i.e., genetics), macroeconomic policies (i.e., access to material goods and health promotion services), and/or historical processes (i.e., colonialism, slavery, immigration, and migration). However, cities can and should outline goals and strategies to become age-friendly cities, through planning healthcare expenditures and attracting investments to the municipality or its metropolitan region. The larger the population of elderly people who remain active and independent for their daily activities the lower the costs will be for long-term treatments for both families and society. Therefore, making cities more friendly to the elderly people is a necessary and logical response, promoting their well-being and keeping these cities prosperous; thus, it is an imperative task force to engage more cities in the WHO program at all levels of government/administration and the participation of all the citizens in the societies.
